# Using tsunami deposits to determine the maximum depth of benthic burrowing

**DOI:** 10.1371/journal.pone.0182753

**Published:** 2017-08-30

**Authors:** Koji Seike, Kotaro Shirai, Naoko Murakami-Sugihara

**Affiliations:** Atmosphere and Ocean Research Institute, University of Tokyo, Kashiwa, Chiba, Japan; Auckland University of Technology, NEW ZEALAND

## Abstract

The maximum depth of sediment biomixing is directly related to the vertical extent of post-depositional environmental alteration in the sediment; consequently, it is important to determine the maximum burrowing depth. This study examined the maximum depth of bioturbation in a natural marine environment in Funakoshi Bay, northeastern Japan, using observations of bioturbation structures developed in an event layer (tsunami deposits of the 2011 Tohoku-Oki earthquake) and measurements of the radioactive cesium concentrations in this layer. The observations revealed that the depth of bioturbation (i.e., the thickness of the biomixing layer) ranged between 11 and 22 cm, and varied among the sampling sites. In contrast, the radioactive cesium concentrations showed that the processing of radioactive cesium in coastal environments may include other pathways in addition to bioturbation. The data also revealed the nature of the bioturbation by the heart urchin *Echinocardium cordatum* (Echinoidea: Loveniidae), which is one of the important ecosystem engineers in seafloor environments. The maximum burrowing depth of *E*. *cordatum* in Funakoshi Bay was 22 cm from the seafloor surface.

## Introduction

The mixing of seafloor sediments by benthic organisms, or bioturbation, is particularly important in marine environments because it influences the biogeochemistry of seafloor deposits [1–3] and disturbs the stratigraphic order [4–6]. The maximum depth of sediment biomixing is directly related to the vertical extent of post-depositional environmental alteration in the sediments [1, 7]; consequently, it is important to improve our understanding of the maximum burrowing depth.

The examination of sediment cores enables detailed observations of the physical and biogenic sedimentary structures preserved within deposits, thereby providing important information on the vertical distribution of these structures [5, 6, 8, 9]. However, sedimentary structures do not necessarily reveal current burrowing activity, as we cannot distinguish current bioturbation structures from those produced in the past (i.e., pre-existing burrows).

Event deposits, or records of event sedimentation such as volcanic ash layers, tempestites (storm deposits), and turbidites may provide information on the nature of bioturbation [10–14]. These event beds allow accurate estimation of the depth of current bioturbation, because there are no pre-existing burrows in the sediments.

**Fig 1:**
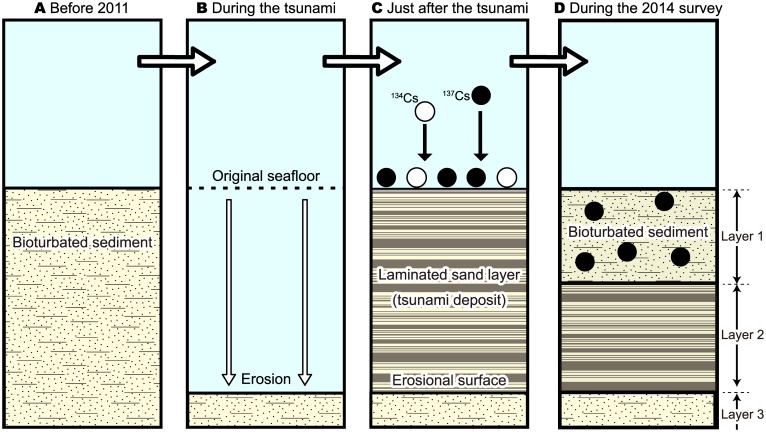
Schematic representation of changes in sedimentary features in an area affected by the 2011 tsunami from before the tsunami to several years after the event. (A) The seafloor situation before the 2011 tsunami. The substrate was bioturbated. (B) Situation during the tsunami. The seafloor was eroded by the strong tsunami current. (C) Just after the 2011 tsunami. The seafloor is covered with tsunami deposits. The radiocesium (^134^Cs and ^137^Cs) released from the Fukushima Nuclear Accident was deposited on the seafloor surface. (D) Expected seafloor situation in the years following the 2011 tsunami. Bioturbated layer lacks physical sedimentary structures and carries the radiocesium due to vertical biomixing of the sediments following recolonization by benthic fauna. Layer 1: tsunami deposits, bioturbated after recolonization by benthic fauna after the event. Layer 2: unbioturbated tsunami deposits, showing well-defined physical sedimentary structures such as parallel laminations. Layer 3: pre-tsunami deposits.

A M9.0 megathrust earthquake (the Tohoku-Oki earthquake) occurred on 11 March 2011 and ruptured the plate boundary off the Pacific coast of northeastern Japan [15]. This earthquake generated a tsunami that affected a 2000 km stretch of the Pacific coast of Japan. Previous papers have described the tsunami inundation and run-up along the Pacific coast [16, 17]. The disturbance caused by the tsunami wave led to the deposition of sandy layers (tsunami deposits) in shallow marine and continental shelf settings along the northeastern Pacific coast of Japan [5, 6, 18–20]. Some benthic animals were found to be absent from the sandy and muddy seafloor ecosystems just after the 2011 tsunami, but began recolonizing in the years following the disaster [21–23]. That is, the 2011 tsunami deposits were bioturbated only by benthic animals recolonizing the seafloor sediments after the event (Fig 1D). This situation provides a rare opportunity to determine an accurate depth for bioturbation in natural marine environments.

The 2011 earthquake and associated tsunami also disrupted the operation of the Fukushima No. 1 Nuclear Power Plant, which resulted in the widespread release of large amounts of radiocesium (^134^Cs and ^137^Cs), as well as other radionuclides, into the local environment over the period 12–23 March 2011. Although the release of these radioactive elements led to contamination of the terrestrial and marine environments (Fig 1C; [24, 25]), it also provided the opportunity to trace the processing of seafloor deposits that occurred after the 2011 earthquake [18–20, 26]. This is because we can use the radioactive elements released from Fukushima to measure the extent of vertical mixing by benthic organisms of the seafloor sediments (Fig 1D; [27, 28]).

**Fig 2:**
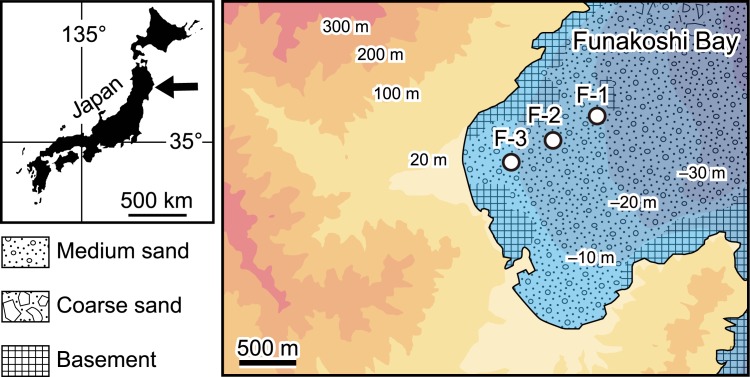
Map of the study area showing the locations of sampling sites.

We investigated the maximum depth of bioturbation in a natural marine environment by observing bioturbation structures developed in an event layer (the 2011 tsunami deposits) and by measuring the radioactive cesium concentrations of marine sediments in Funakoshi Bay, northeastern Japan (Fig 2). In addition, we consider which of the two methods provides the most accurate depth of bioturbation.

The spatangoid echinoid *Echinocardium cordatum* (Pennant, 1777), which is an important bioturbator in coastal ecosystems, inhabits the sandy bottom of Funakoshi Bay. Bioturbation by *E*. *cordatum* affects not only the biogeochemical properties of the sediments, but also the nutrient flux from substrate to water column [3, 29, 30]. Revealing the depth of echinoid burrowing is important if we are to better understand the effects of bioturbation on benthic ecosystem functioning. This echinoid inhabited the seafloor sediments of Funakoshi Bay before the 2011 tsunami, but was absent in 2011 following the tsunami, before then reappearing in 2012 [21]. This re-establishment of the spatangoid echinoderm population must also represent the point in time at which bioturbation activity restarted after the tsunami. Therefore, the tsunami deposits have been bioturbated mainly by the recolonized *E*. *cordatum*, and so can be used to obtain an accurate estimate of the burrowing depth of this species.

The heart urchin *E*. *cordatum* produces a specific biogenic structure in the sediment, which can be preserved as a trace fossil in the geological record [31–37]. Because knowledge of this structure contributes not only to marine biology but also to paleontology, we investigated it in detail.

## Materials and methods

### Ethics statement

The field survey described in this study was approved by the local fisheries cooperative and the government of Iwate Prefecture.

### Study sites

Funakoshi Bay is located on a ria coast in northeastern Japan (Fig 2). The bay faces the Pacific Ocean and is affected by direct oceanic wave activity, with no river outlets or wide coastal plains. At water depths of less than 20 m, the seafloor is covered mainly by fine-grained sandy deposits [21, 38, 39]. Sampling sites F-1, F-2, and F-3 were in water depths of about 20, 15, and 10 m, respectively (Fig 2; Table 1).

**Table 1:** Location, water depth, and median grain size of surface sediment at each coring site.

Site	Latitude (N)	Longitude (E)	Water depth (m)	Median grain size (mm)
F-1	39°23.250'	141°57.109'	20.0	0.184
F-2	39°23.135'	141°56.861'	15.8	0.179
F-3	39°23.036'	141°56.611'	10.0	0.171

### Impact of the 2011 tsunami on benthic ecosystems in Funakoshi Bay

The extent of the inundation and run-up along the Pacific coast associated with the 2011 tsunami has been described by Mori et al. [16] and The 2011 Tohoku Earthquake Tsunami Joint Survey Group [17]. The narrow bays along the ria coast focused the tsunami waves, generating high inundation and run-up. The behavior of the tsunami on land varied among regions, even between the adjacent bays. At Funakoshi Bay, the maximum height of tsunami inundation and run-up were 22.1 and 29.3 m, respectively, and the maximum run-up distance was 1.1 km from the coastline.

Our study sites were located in the inner part of the embayed coast (Fig 2), where the seafloor topography is not normally affected by ocean waves. In contrast to the normal situation, the existing seafloor sediments were strongly disturbed by the 2011 tsunami and were then covered with a new layer of tsunami deposits (Fig 1C; [21]). Major infaunal animals, including *E*. *cordatum*, were no longer present just after the event due to the disturbance. On the other hand, Seike et al. [21] also investigated the changes in seafloor topography, sediment grain size, and benthic animal assemblages in Funakoshi Bay following the 2011 tsunami. Seafloor topography and sediment grain size showed little change after the event.

### Sampling of *Echinocardium cordatum*

**Fig 3:**
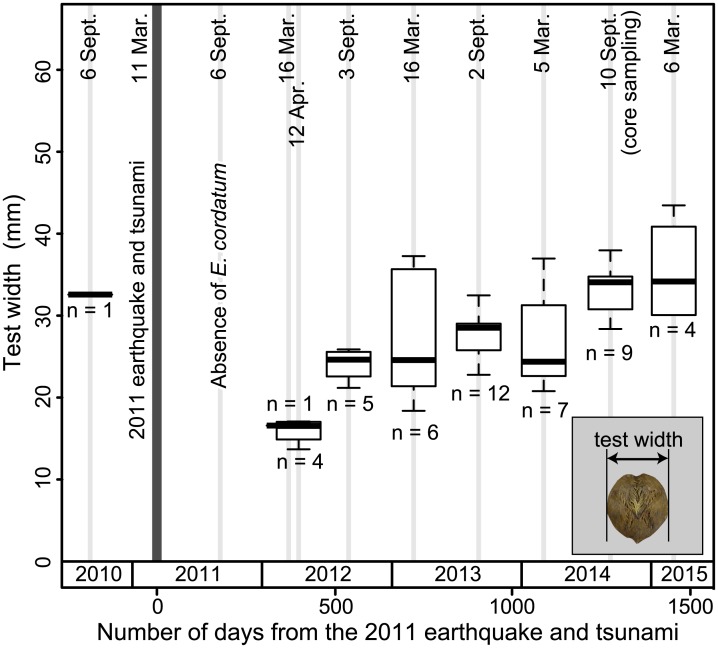
Temporal change in test width of *Echinocardium cordatum* in Funakoshi Bay before and after the 2011 tsunami. The *E*. *cordatum* population disappeared in 2011 and began to recolonize from early 2012. The test width had reached the same size as before the tsunami by September 2014, when the sediment cores were collected.

The seafloor sediment samples were retrieved using the Smith—McIntyre grab sampler (one grab sample in March and April of 2012; five grab samples in September of 2012–2014, and March of 2013–2015), and then sieved through a 1-mm mesh screen to retrieve *E*. *cordatum*. The test width (Fig 3) was measured using a caliper to the nearest tenth of a millimeter. Sampling was conducted at all three sites (F-1, F-2, and F-3) in March and September over the period 2012–2015 to monitor any temporal change in test width of *E*. *cordatum*. In addition, scuba diving was used to record the presence or absence of *E*. *cordatum* in the bay.

### Sampling of sediment cores

Scuba divers collected sediment cores (6 cm diameter, 100 cm length) in September 2014. The sedimentary structures preserved within the cores were examined using a computed tomography (CT) scanner (LightSpeed Ultra16, GE Healthcare Japan Corp.) at the Center for Advanced Marine Core Research, Kochi University, Kochi, Japan. The CT scans were visualized using OsiriX imaging software (version 4.1.2), which allowed us to observe both physical and biogenic sedimentary structures in the core [5,6]. After CT scanning, the cores were sliced into 5-cm-long subsamples for analysis of the grain-size distribution and radioactivity of cesium isotopes. The grain-size distribution of the subsamples was analyzed using a laser granulometer (Mastersizer 2000, Malvern Instruments) at the Center for Advanced Marine Core Research, Kochi University, Kochi, Japan. The radioactivity of the cesium isotope ^137^Cs in the subsamples was determined by γ-ray spectrometry using a well-type germanium detector (Canberra GCW3523) at an ultra-low background laboratory in the Institute for Cosmic Ray Research, the University of Tokyo, Kashiwa, Japan. Gamma-ray emission at an energy of 662 keV was measured for between 46 and 263 hours depending on the level of radioactivity. Detection limit was largely determined by duration of measurement and were approximately 0.7 and 1.3 bq/kg for 263 and 46 hours, respectively. Efficiency correction was performed based on the IAEA-444 soil standard. We continued to measure the radioactivity until the core depth was reached that showed a ^137^Cs concentration below the detection limit. Short-lived radionuclides, such as ^131^I and ^134^Cs, were not detected because the radioactivity analysis was conducted after 2015; i.e., more than 4 years after the accident at Fukushima.

**Fig 4:**
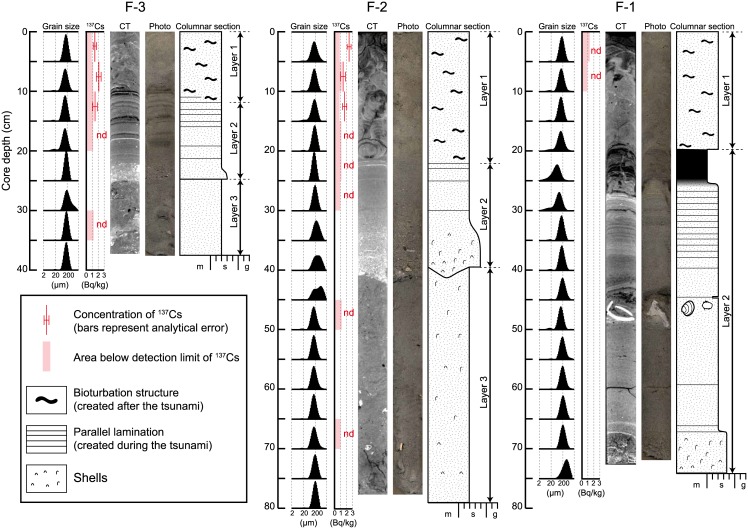
Grain-size distributions, concentrations of ^137^Cs, CT images, photographs, and columnar sections for the cores. Layer 1, the upper part of the cores, was bioturbated. Layer 2, the sediments between the base of Layer 1 and the erosional surface (the coarse-grained bed), shows well-defined physical sedimentary structures such as parallel laminations. Layer 3, the sediments beneath the erosional surface, show neither physical sedimentary structures nor obvious bioturbation structures. nd: not detected. m: mud. s: sand. g: gravel.

**Fig 5:**
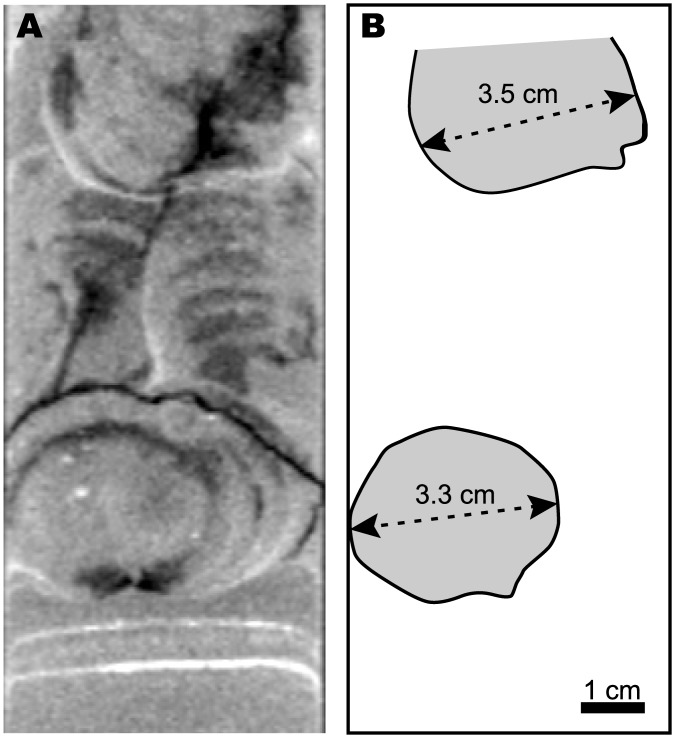
Close-up view of the burrows produced by *Echinocardium cordatum*. (A) CT image of the upper part of core F-2. (B) Sketch of (A). The transverse width of the *E*. *cordatum* burrows is ~3 cm.

Bioturbation structures produced by *E*. *cordatum* were seen on the CT image of the all cores and were especially evident on the image of core F-2 (Figs 4 and 5). The transverse width of the burrow structure was measured to compare with that of the producer (test width of *E*. *cordatum*).

## Results

### Temporal change in body size of *Echinocardium cordatum*

During the study period, the spatangoid echinoid *E*. *cordatum* was found at all three sampling sites in Funakoshi Bay, except during the period immediately after the 2011 tsunami. Only a single specimen of *E*. *cordatum* was collected before the 2011 tsunami, and the test width was 32.6 mm. The median test width increased with time after the 2012 sampling period (Fig 3). In September 2014, the width was similar to that before the 2011 event.

### Sediment cores

Grain-size analysis revealed that the seafloor is composed mainly of fine-grained sandy deposits, although some coarser-grained and muddy layers were also identified (Fig 4).

Based on the sedimentary structures, we divided the cores into three lithological layers (Figs 1 and 4). Layer 1, the upper part of the cores, was bioturbated and lacked any physical sedimentary structures. This layer was composed of fine-grained sands. Layer 2, which represents the sediments between the lower part of Layer 1 and the base of the coarse-grained bed, was not bioturbated and contained well-defined physical sedimentary structures such as parallel laminations. This layer was composed of fine- to coarse-grained sands with pebble-sized clasts and molluscan shells. Layer 3, which represents the sediments beneath the coarse-grained bed, contained neither physical sedimentary structures nor obvious bioturbation structures. This layer was composed of fine-grained sands. Layer 3 occurred in cores F-2 and F-3, but not F-1. The sequence of these layers was the same in all cores; however, the thickness of Layer 1 (depth of bioturbation from the seafloor) differed among the sampling sites, being 19, 22, and 11 cm at sites F-1, F-2, and F-3, respectively (Fig 4). On the CT image of core F-2, well-defined burrow structures produced by *E*. *cordatum* were evident (Fig 4), showing a transverse width of ~3 cm (Fig 5).

The concentration of ^137^Cs in the uppermost section of the cores (depth: 0–5 cm) also differed among the sampling sites (Fig 4). ^137^Cs was detected in cores F-2 and F-3, but not F-1. The base of the ^37^Cs layer was at a depth of 10–15 cm in cores F-2 and F-3.

## Discussion

We interpret layers 1 and 2, the sediments above the coarse-grained bed, as tsunami deposits. The rationale for this interpretation is as follows. The seafloor of our study site in Funakoshi Bay is normally a low-energy (hydrodynamically calm) environment (10–20 m deep), where the sandy deposits of the seafloor would not normally be affected by ocean waves or storms. On the other hand, the parallel laminations in Layer 2 suggest that deposition occurred under conditions of strong bottom water currents that rarely occur at these sites. We conclude that parallel laminations were deposited by the strong current related to the 2011 tsunami. In fact, parallel laminations are one of the major criteria for the recognition of tsunami deposits [40]. Hence, we interpret the bottom of Layer 2 (coarse-grained bed) as the erosional surface created by the tsunami current, and Layer 3 as pre-tsunami deposits, because this layer is overlain by deposits of the 2011 tsunami. Layer 3 does not contain any clear physical or biogenic sedimentary structures, which may have resulted from liquefaction associated with the earthquake [41].

The seafloor of Funakoshi Bay is normally a hydrodynamically calm environment. Therefore, changes in seafloor topography are rare. In fact, the seafloor topography (water depth) of each site in the 2014 survey was almost the same as that recorded in 2012 by Seike et al. [21]. The thickness of Layer 1 (bioturbated sediment), therefore, represents the true depth of bioturbation at each sampling site, because no significant aggradation or erosion of the seafloor occurred at these sites after the tsunami event.

Our interpretations are also supported by the sizes of the echinoid burrows. The transverse width of the burrow in the F-2 core is similar to the test width of its producer (*E*. *cordatum*) at the time of sampling (September 2014); i.e., the test of the echinoid had reached this width for the first time since the tsunami (Fig 3). If the seafloor had aggraded upwards due to sedimentation, small burrows produced by the juvenile echinoids that recolonized in early 2012 should have been evident in the cores; however, such small burrows were not present in the core samples. Burrowing depth of juvenile echinoids should have been less than that of adult echinoids, indicating that the subsequent large burrows in 2014 overprinted and obliterated the small burrows produced in early 2012. Therefore, the burrows in core F-2 represent the current extent of bioturbation; i.e., the depth of ongoing bioturbation as at September 2014.

The core data also show that the depth of bioturbation (i.e., the thickness of Layer 1) varies among the sampling sites, even in this one bay. The burrowing depth of irregular echinoids is associated with sedimentological (e.g., grain size composition) and biogeochemical (depth of the redox potential discontinuity layer) characteristics of the seafloor sediment [42, 43]. However, our data cannot reveal the cause of this variation because we have cores from only three sites. If we are to reveal the nature of depth variations in bioturbation in the study area, it will be essential for future quantitative studies to obtain cores from many more sampling sites across the bay.

Theoretically, the lower limit of ^137^Cs detection should correspond to the base of Layer 1 (Fig 1D), as it did, in fact, in core F-3. However, for core F-2, ^137^Cs was detected only to a core depth of 10–15 cm, which is half the thickness of Layer 1. These observations indicate that the maximum depth of ^137^Cs distribution differs from the vertical range of bioturbation. Furthermore, no ^137^Cs was detected even in the seafloor surface sediment at site F-1, indicating that the processing of ^137^Cs in coastal environments contains extra pathways (e.g., lateral transportation process) in addition to bioturbation.

*Echinocardium cordatum* is an important bioturbator and has therefore been widely studied. However, the extensive literature shows remarkable differences in observations and interpretations of the structures produced by this species (summarized by [36]). Based on direct observations in the laboratory and field, *E*. *cordatum* is known to be able to burrow to a depth of 10–20 cm from the sediment surface [31, 32, 44]. However, the results from laboratory experiments differ from those from natural marine environments. For example, it is difficult to precisely recreate the settings of sediment porosity, firmness, and water content in aquarium experiments. In addition, measuring accurate burrowing depths is also difficult when observing animals in the field. Hence, the above methods yield only rough estimates of the actual burrowing depth of *E*. *cordatum*. On the other hand, experimental field measurements made using a fluorescent tracer showed limited downward particle movement (to a depth of 5 cm), indicating that *E*. *cordatum* burrows to a depth of 5 cm [45]. However, the maximum depth of particle movement does not directly correspond to that of bioturbation. For these reasons, the maximum depth of echinoid bioturbation in natural marine environments remains unclear.

The present study has revealed the maximum burrowing depth of *E*. *cordatum* by observing the burrow structures that were made in an event deposit (i.e., the 2011 tsunami deposits). The core obtained from site F-2 shows the vertical distribution of the bioturbation structures created by *E*. *cordatum*, indicating that this echinoid can burrow to a depth of 22 cm from the seafloor in the natural environment. This finding corresponds closely to the maximum burrowing depth of *E*. *cordatum* measured by aquarium experiments and field observation in previous studies [31, 32, 44], but the significance of our result is that it is a natural and realistic value obtained in the field. The F-2 core provides the first clear evidence that *E*. *cordatum* can burrow at a depth of >20 cm below the sediment surface in the natural marine environment. Investigating bioturbation structures in event deposits, such as tsunami and storm layers, will help us to better understand the nature of bioturbation, in particular the maximum burrowing depth of benthic animals.
